# A protocol for the development of core outcome sets for effectiveness trials and clinical audits in renal cell cancer (R‐COS)

**DOI:** 10.1002/bco2.266

**Published:** 2023-06-28

**Authors:** Steven MacLennan, Lisa M. Wintner, Katharina Beyer, Ailbhe Lawlor, Sheela Tripathee, Saeed Dabestani, Lorenzo Marconi, Rachel H. Giles, Rose Woodward, Mieke Van Hemelrijck, Axel Bex, Patricia Zondervan

**Affiliations:** ^1^ Academic Urology Unit, Institute of Applied Health Sciences University of Aberdeen Aberdeen UK; ^2^ University Hospital of Psychiatry II Medical University of Innsbruck Austria; ^3^ Translational Oncology and Urology Research (TOUR) King's College London London UK; ^4^ Department of Translational Medicine, Division of Urological Cancers Lund University Lund Sweden; ^5^ Department of Urology Kristianstad Central Hospital, Region Skane Kristianstad Sweden; ^6^ Department of Urology Coimbra University Hospital Coimbra Portugal; ^7^ International Kidney Cancer Coalition Duivendrecht The Netherlands; ^8^ Action Kidney Cancer Charity Manchester UK; ^9^ Department of Urology Amsterdam University Medical Centers Amsterdam The Netherlands; ^10^ Specialist Centre for Kidney Cancer Royal Free Hospital London UK; ^11^ Division of Surgery and Interventional Science University College London London UK

**Keywords:** consensus methods, core outcome set, Delphi, evidence‐based medicine, nephrectomy, renal cell cancer

## Abstract

**Background:**

There is inconsistency in outcomes collected in renal cell cancer (RCC) intervention effectiveness studies and variability in their definitions. This makes critical summaries of the evidence base difficult and sub‐optimally informative for clinical practice guidelines and decision‐making by patients and healthcare professionals. A solution is to develop a core outcome set (COS), an agreed minimum set of outcomes to be reported in all trials in a clinical area.

**Objectives:**

To develop three COS for (a) localised, (b) locally advanced and (c) metastatic

**RCC study design, participants and methods:**

The methods are the same for each of our three COS and are structured in two phases. Phase 1 identifies potentially relevant outcomes by conducting both a systematic literature review and patient interviews (N ~ 30 patients). Qualitative data will be analysed using framework analysis. In phase 2, all outcomes identified in phase 1 will be entered in a modified eDelphi, whereby patients and healthcare professionals (50 of each) will score each outcome's importance (Likert scale from 1 [not important] to 9 [critically important]). Outcomes scored in the 7–9 range by ≥70% and 1–3 by ≤15% will be regarded as ‘consensus in’, and the vice versa of this will constitute ‘consensus out’. All other combinations will be regarded as equivocal and discussed at consensus meetings (including 10 patients and 10 healthcare professionals) in order to vote on them and ratify the results of the eDelphi.

**Discussion:**

The R‐COS will reduce outcome reporting heterogeneity and improve the evidence base for RCC.

**Study registration:**

The study is registered with the COMET initiative: https://www.comet-initiative.org/studies/details/1406.

## 1 BACKGROUND

Renal cell cancer (RCC) accounts for 2%–3% of all malignancies globally.^1^ The incidence is increasingly common in Western societies.^2^ Mortality rates vary significantly globally and are decreasing in some Scandinavian countries, as well as France, Germany, Austria, the Netherlands and Italy, whereas it is rising in others (e.g., Ireland, Croatia, Greece, Estonia and Slovakia).^3^ Risk factors include smoking, obesity, hypertension and chronic kidney disease.^2^, ^4^

There are several subtypes of RCC, of which clear cell, papillary and chromophobe are the most frequent occurring.^5^ All RCCs are, however, based on tumour size and level of dissemination, categorised as localised, locally advanced or metastatic—each stage indicating a worsening prognosis. In the localised group, treatment intent is curative, usually with a partial or radical nephrectomy, but other options such as cryotherapy, radiofrequency ablation or active surveillance are also offered.^6^ Various surgical, tyrosine kinase inhibitor and immunotherapy treatments are available for metastatic disease.^6^ The treatments have different benefit‐to‐harm profiles, which means that optimal treatment choice is difficult for patients and health care professionals. One reason for uncertainty is outcome reporting heterogeneity in RCC trials,^7^, ^8^, ^9^, ^10^, ^11^ which refers to an interrelated group of problems: inconsistency (different effectiveness trials report different outcomes), variability (the same outcomes are reported but are measured and/or defined differently) and selective outcome reporting (outcomes with statistically significant results are more likely to be reported).^12^ We recently published a systematic review of outcome reporting in localised RCC intervention effectiveness studies (phase 1 of the project outlined in the current manuscript) and found outcome reporting inconsistency and variability.^13^ For example, adverse events were commonly reported, but this was not standardised across studies. Some reported discrete events at time points that differed across studies; others used systems like Clavien–Dindo, which focus on the consequence of the event rather than the event itself; whereas others used ‘trifecta’ or ‘pentafecta’ collations of outcomes to provide a summary. These ways of expressing adverse events are non‐commensurable when it comes to narratively or statistically synthesising the evidence base. While researchers could technically extract individual events and recode them to a standard system such as Clavien–Dindo, the process would be inefficient and laborious. Quality of life (QoL) was, worryingly, only reported in 3/145 (2%) of the included studies, and different tools were used in each. Little can be done to make these tools commensurate, and an analogous solution to the adverse event recoding situation provided above would not be possible. Of note, we did not search for protocols and trial registrations for each of the 149 included studies to assess selective outcome reporting due to resource constraints, so it is not possible to say presently if and to what extent this methodological issue is present in the localised RCC evidence base.

Outcome reporting inconsistency, variability and selective outcome reporting obscure the interpretation of intervention effectiveness, which means meta‐analysis in systematic reviews is often ill‐advised, or worse, done regardless, and potentially provides misleading estimates. Subsequently, clinical practice guideline panels are left with unwieldy narrative summaries, which precludes them from providing strong and definitive recommendations on the relative benefits and harms of various treatments.^14^, ^15^ Therefore, there is an urgent need in the international urology and oncology communities to identify those outcomes that are the most important to all stakeholders and ought to be reported in all RCC trials and audits concerning interventions for the various stages of RCC.

A solution to outcome reporting heterogeneity is a core outcome set (COS), which is an agreed minimum set of outcomes to be reported in all trials in a clinical area.^14^ In developing COS, it is important to include the opinions of key stakeholders to prioritise what the most important outcomes are from their perspective.^14^, ^16^, ^17^ Given their experiences as givers or receivers of care, patients and healthcare professionals are the key stakeholders in the development of the Renal cell cancer – Core Outcome Sets (R‐COS).

The developed COS can be used for effectiveness trials, systematic reviews, clinical practice guidelines and routine clinical audits. Hence, the COS is fit for evaluating therapies as well as auditing any interventions with experimental conditions, enabling benchmarking. The audit can also enable surveillance of practice patterns in the context of implementation.^18^ A further benefit of using COS is the enhanced information value of the data for big data projects, as outcome definitions and their measurement are standardised across clinical trials and everyday clinical care.

The R‐COS project will standardise outcome reporting, facilitate critical appraisal and evidence synthesis, and optimise the use of resources. Ultimately, this will lead to an increase in the strength of the intervention effectiveness evidence base, improve the robustness of clinical practice guideline recommendations, and facilitate decision‐making for patients, healthcare payers, healthcare policy makers and clinical practice.

## 2 OBJECTIVES

Our aim is to create a COS, which is defined as ‘an agreed standardised collection of outcomes which should be measured and reported, as a minimum, in all trials for a specific clinical area’.^14^ We aim to create one COS each for (a) localised, (b) locally advanced and (c) metastatic RCC. The target population is male and female adults (>18) with a diagnosis of localised, locally advanced or metastatic RCC. Given the different prognoses and interventions for each stage of the disease, it is sensible to treat them as separate COS but also to coordinate them within a broader project. There are published standards for the development and reporting of COS,^19^, ^20^ and our overall project design follows this guidance. We report our protocol in line with the COS‐standard protocol items (COS‐STAP).^21^

Our specific objectives are:

1. To systematically review the literature for effectiveness trials of interventions for (a) localised, (b) locally advanced and (c) metastatic RCC and extract the outcomes they report
2. To conduct semi‐structured interviews with RCC patients in order to investigate patient experiences of the disease and its treatments and to better understand the range of important outcomes from the patient perspective
3. To merge data from literature and patient interviews and create an exhaustive list of relevant outcomes for (a) localised, (b) locally advanced and (c) metastatic RCC
4. To conduct, for each outcome list, individual online modified Delphi surveys (eDelphi) in order to let key stakeholder groups rate the importance of listed outcomes
5. To convene consensus meetings with representatives from the key stakeholder groups to vote on and ratify each R‐COS
6. To disseminate the R‐COS for (a) localised, (b) locally advanced and (c) metastatic RCC

We recognise that there may be instances where some measures are not feasible or acceptable in everyday settings and will endeavour to recommend definitions and measurements dependent on setting in these instances; this will come as part of the later ‘how to measure’ phase of our research.

We will encourage international participation in the identification and prioritisation of the outcomes through our international networks (for example, members of our project steering group sit on the European Association of Urology [EAU] RCC guideline panel, one directs the International Kidney Cancer Coalition [IKCC], and others are involved in the European Organisation for Research and Treatment of Cancer [EORTC] cancer QoL tool development programme). In disseminating the R‐COS, we will emphasise that outcomes other than those deemed core may also be measured, so long as those deemed core are measured as a minimum. We will also engage with journal editors and the Cochrane Urology group, as well as research funders and guideline organisations (such as the EAU, British Association of Urological Surgeons [BAUS] and American Urological Association [AUA]), to encourage researchers to use the R‐COS or to justify why it is not relevant to their study, as part of our dissemination and implementation strategy.

## 3 METHODS

The recommendations from the Core Outcome Measures in Effectiveness Trials (COMET) and the COnsensus‐based Standards for the selection of health Measurement INstruments (COSMIN) will be followed in order to identify the COS (the ‘what’ to measure) and the relevant outcome measurement instruments (OMI, the ‘how’ to measure).^14^, ^22^

The project steering group consists of 10 members (SM, LW, KB, MVH, LM, SD, AB, PZ, RG and RW) who have expertise in COS development methods (SM, KB and MVH), RCC QoL tool development (LW), RCC patient management, research and guideline development (AB, LM, SD and PZ), and we have also included an expert patient (RG, founder of the International Kidney Cancer Collaboration [IKCC]) and a patient advocate (RW, co‐founder of Action Kidney Cancer) in order to strengthen the integration of patient perspectives into R‐COS development through advocacy representatives. This approach improves the inclusivity of the project and ensures that the materials developed are appropriate for the RCC community.

Appropriate applications will be made to a UK research ethics committee for the interview, the online Delphi (eDelphi) process and consensus meeting studies.

## 4 STUDY DESIGN

The same study design and methodology will apply to the development of COS for (a) localised, (b) locally advanced and (c) metastatic RCC. In phase 1, we will generate a list of outcomes currently reported in the literature through systematic reviews of the effectiveness of RCTs. We will supplement the list identified from systematic reviews with a descriptive cross‐section design interview study, including a sample of patients who have been diagnosed with RCC and have undergone treatments.

In phase 2, we will include the list of outcomes identified in phase 1 in a cross‐section design eDelphi process: one for each defined RCC subgroup. We aim to lessen the burden for healthcare professional involvement by leaving at least 6 months between the individual Delphi processes for each R‐COS because. Although urologists may deal more often with localised disease, and oncologists contribution becomes more involved in the more advanced stages, it will largely be the same healthcare participants across the three R‐COS Delphi projects. This may mitigate against a low response rate during the Delphi survey.^23^ Patient participants will be different for each stage.

**FIGURE 1:**
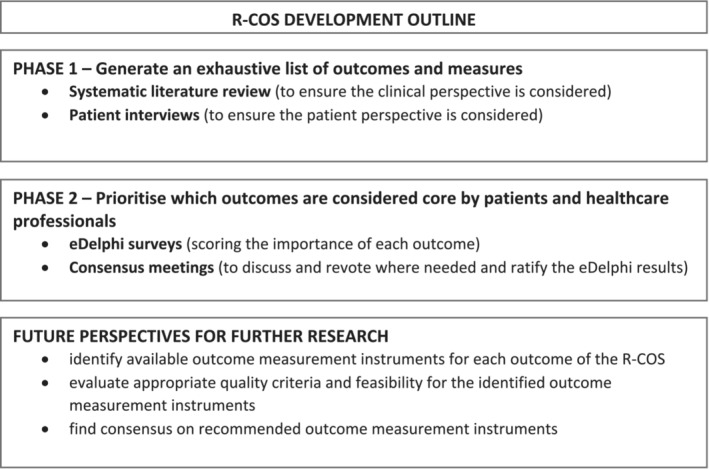
Renal cell cancer – Core Outcome Sets (R‐COS) development outline.

The study plan is outlined in Figure 1.

### 4.1 Phase 1: generation of a list containing all potentially relevant outcomes

#### 4.1.1 Systematic reviews

We will perform three systematic reviews, one each for (a) localised, (b) locally advanced and (c) metastatic RCC. These reviews will aim to survey the outcomes that have been reported in intervention effectiveness trials. We have separately published the protocol for localised disease (https://www.crd.york.ac.uk/prospero/display_record.php?RecordID=198605). The other two reviews followed the same procedure and were adjusted accordingly.

#### 4.1.2 Search terms and search strategy

Our search strategy will use MeSH terms and will be tailored to the populations and interventions for each of the three disease stages outlined. We will search Medline, Embase and the Cochrane Controlled Register of Trials (CENTRAL), as the combination of these databases has been shown to be the most appropriate for reviews of intervention effectiveness.^24^ No language restrictions will apply. We will search the most recent 5 years initially, extract data, then move iteratively back in time by one‐year increments until no ‘new’ outcomes are identified. This strategy allows us to ensure our results reflect current practice and balance comprehensiveness with reviewer burden. Two authors will independently screen the abstracts and full texts. Disagreements will be resolved by a third review author.

#### 4.1.3 Types of studies included

From our groups' various prior systematic reviews and guideline development work in this field^6^, ^7^, ^8^, ^9^, ^10^, ^11^ and scoping searches, it is known that many comprehensive intervention effectiveness systematic reviews already exist for the interventions listed above. Therefore, for the quantitative systematic reviews, we will restrict them to RCTs and comparative observational studies included in systematic reviews only. All other study designs will be excluded.

#### 4.1.4 Types of patients included

The populations and interventions for each review differ, and indeed, the interventions in scope for each of the three COS according to disease stage are outlined in Table 1.

**TABLE 1:** List of included interventions in the systematic literature research.

Localised RCC (defined as up to stage T2b N0 M0)	Locally advanced RCC (defined as any N+ or T3‐T4 N0 M0)	metastatic RCC (defined as Tany N+ M+)
Active surveillance		
Radical nephrectomy (any technique: open, laparoscopic, robot‐assisted, transperitoneal, retroperitoneal, etc.)	Radical nephrectomy (any technique: open, laparoscopic, robot‐assisted, transperitoneal, retroperitoneal, etc.)	Cytoreductive nephrectomy; Partial nephrectomy (any technique: open, laparoscopic, robot‐assisted, transperitoneal, retroperitoneal, etc.)
Partial nephrectomy (any technique: open, laparoscopic, robot‐assisted, transperitoneal, retroperitoneal, etc.)	Partial nephrectomy (any technique: open, laparoscopic, robot‐assisted, transperitoneal, retroperitoneal, etc.)	Partial nephrectomy (any technique: open, laparoscopic, robot‐assisted, transperitoneal, retroperitoneal, etc.)
		Immunotherapy
Adjuvant systemic therapy (targeted therapies, immunotherapy)	Adjuvant systemic therapy (targeted therapies, immunotherapy)	Targeted therapies
		Systemic therapy (in 1st line, 2nd line, 3rd line, etc.)
Cryoablation		
Radiofrequency ablation		Radiotherapy (any type or dose)
Other ablative techniques (microwave, laser, HIFU)		
Radiotherapy (stereotactic ablative body radiotherapy [SABR])	Radiotherapy (SABR)	
Associated procedures: adrenalectomy, embolisation	Associated procedures: LND, adrenalectomy, inferior vena cava (IVC) tumour thrombectomy	
		Metastasectomy

Abbreviation: RCC, renal cell cancer.

#### 4.1.5 Data extraction and analysis plans

Two authors will independently extract data from included full texts using a data extraction form. Disagreements will be resolved by a third review author. Outcomes relating to intervention effectiveness will be extracted and recorded in a Microsoft Excel file. We will record which outcome is designated as the primary outcome, then extract the following data for all reported outcomes: verbatim outcome name, definition of the outcome, timing and measurement of the outcome, and the method used to express the outcome (for example, percentages at median follow‐up or time to event hazard ratios—because these also have importance for deciding whether outcomes can be combined in meta‐analyses and give us an idea of heterogeneity). After assessing heterogeneity, we will code conceptually similar outcomes, regardless of the exact definition, to common outcome names and further organise the outcomes under domains using the Williamson/Clarke Taxonomy.^25^

Risk of bias will not be assessed, as this is not applicable when identifying outcomes, as we aim to do in identifying the R‐COS.

The list of outcomes identified in the systematic reviews will be included in the eDelphi survey.

#### 4.1.6 Semi‐structured interviews with patients

To adequately include the patient perspective, we will conduct semi‐structured interviews with a sample of English‐speaking people who are diagnosed with RCC and received treatment. We will apply a purposeful sampling strategy to adequately represent males and females, the various types of treatments available and the spectrum of disease stages. To achieve data saturation, the data will be analysed after the inclusion of eight patients, and further sets of each of the three patient interviews will be conducted until no more new data emerges.^26^ We will recruit patients through our patient and public involvement (PPI) partners' networks. We will use a framework approach to analysis,^27^ coding themes across interviews into common outcome names. Data management and analysis will be aided using QSRNVivo.^28^ Any outcomes identified will be added to the list of outcomes for the eDelphi.

Appropriate applications will be made to UK research ethics committee for the interview, eDelphi and consensus meeting studies.

### 4.2 Phase 2: prioritisation of important outcomes from stakeholder groups

#### 4.2.1 Preparation of the outcomes list

The lists of outcomes obtained from the systematic review and the patient interviews will be combined. The study steering group will review the list of outcomes and combine conceptually similar outcomes. We will apply the COMET outcome definition (‘… a measurement or observation used to capture and assess the effect of treatment such as assessment of side effects (risk) or effectiveness (benefits)’^14^) and exclude any items that do not meet that definition. All the outcomes identified in phase 1 (systematic literature review and patient interviews) will be formatted as questionnaire items.

#### 4.2.2 eDelphi process

We will have a separate eDelphi for (a) localised, (b) locally advanced and (c) metastatic RCC, but the process will be identical for each. There will be a maximum of three Delphi rounds. All items will be retained in all Delphi rounds.

The healthcare professionals will be invited via the EAU RCC guideline panel's network and will include urologists, oncologists, radiotherapists, interventional radiologists and cancer nurse specialists. They will be regarded as a homogenous stakeholder group for the Delphi analysis (i.e., we will not analyse differences between these groups). Patients will be invited through IKCC's network and will be regarded as a homogenous group regardless of treatment type in the analysis. The Delphi will be managed entirely online using the DelphiManager software (http://www.comet-initiative.org/delphimanager/). An invitation email will contain a participant information page explaining the study rationale and a link to the eDelphi. Registering for eDelphi and completing it will imply informed consent.

#### 4.2.3 Delphi Round 1

Participants will be asked to score the importance of each outcome on a scale of 1 (not important) to 9 (critically important). Participants will have the opportunity to suggest new outcomes. Any new outcome suggested will be reviewed by the study steering group, and if deemed to not be adequately covered already, it will be added to Round 2. For the purpose of providing feedback in subsequent rounds, analysis will involve calculating the percentage of participants choosing each scoring option on the 1–9 scale. This will be stratified by the stakeholder group.

#### 4.2.4 Delphi Round 2

Participants will be reminded of their own score from Round 1 and shown a summary of the distribution of scores given to each outcome stratified by stakeholder group (i.e., multiple separate feedbacks). There is some evidence to suggest that this strategy is better for achieving consensus.^29^, ^30^ Participants may then choose to retain the same score or to change score.

#### 4.2.5 Delphi Round 3

A third round will only be performed if there is consensus on <85% of the outcomes after Round 2. This has been chosen as there is some evidence to suggest that if agreement in Rounds 1 and 2 is already high, then there is little further gain in consensus in subsequent rounds.^29^

#### 4.2.6 Delphi analysis

We define consensus in this context as the outcomes retained by both stakeholder groups (consensus in) or retained by neither (consensus out). In the final analysis of the Delphi, consensus will be achieved if an outcome is rated as 7 to 9 by ≥70% and ≤15% from 1 to 3 of each stakeholder group independently (‘consensus in’), or if an outcome is scored 7–9 by ≤15% and 1–3 for ≥70% of each stakeholder group independently (‘consensus out’). All the outcomes not covered in the above definitions will be considered ‘equivocal’ and will be discussed at the consensus meeting.

During the eDelphi, participants will be unable to submit their scores if there are any blanks; they will, however, have an ‘unable to score’ option. Therefore, we do not anticipate any missing data. An attrition analysis will be done to ascertain whether participants who completed Round 1 but did not complete Round 2 scored differently from those who completed all rounds of the Delphi. This will be done by comparing the mean scores and standard deviations of completers and non‐completers.

#### 4.2.7 Consensus meetings

There will be separate one‐day consensus meetings for each of the three COS. They will be held on separate occasions but will follow the same format. Participants will be sampled from those who completed all rounds of the Delphi, and a maximum of 20 participants (10 patients and 10 healthcare professionals) will be invited to each consensus meeting. Prior to the meetings, each participant will be emailed a summary of the results from the Delphi and a reminder of how they scored each outcome in each round of the Delphi. The aim of the meeting will be to check on the results of Delphi. The ‘consensus in’ and ‘consensus out’ outcomes will be reported and briefly discussed. The main focus will be to discuss the equivocal outcomes to check the reasoning for divergent (i.e., bimodal distributions in the scores for an outcome) or equivocal (i.e., majority scoring 4–6 for an outcome) opinions. Further voting on the equivocal Delphi items will be done anonymously and managed using Poll Everywhere software (https://www.polleverywhere.com/). The scoring scale and consensus definitions will be the same as in eDelphi. The unit of analysis will be a single heterogeneous panel rather than multiple homogenous stakeholder groups because the aim is to seek consensus on what outcomes should be regarded as core for all stakeholders in future RCC research.

### 4.3 Dissemination plan

Potential barriers to the uptake of R‐COS could relate to a lack of awareness of the COS. We will try to address this through a targeted and multi‐platform active dissemination strategy.

For each of the RCC disease stages, (a) localised, (b) locally advanced and (c) metastatic RCC, an individual report will be written as a journal article and submitted to a urology specific journal. Furthermore, the R‐COS will also be disseminated through presentations submitted to urology‐specific conferences, including the EAU annual congress, the BAUS annual congress and the American Urological Association annual congress.

We intend to promote implementation by writing to urology‐specific journal editors to encourage the use of R‐COS in studies submitted to their journals where appropriate and to encourage researchers to justify why they choose not to use R‐COS. We also anticipate that research funders (e.g., the National Institute of Health Research [NIHR]) will facilitate newly registered RCC trials to use the relevant COS and will make our materials available to facilitate use of the R‐COS. We will also publicise the results through the patient community via our PPI partners to ensure that patients are aware of the R‐COS and to clarify that patients were involved as stakeholders in the research.

Beyond this, in a separate phase of the project, we aim to systematically review the psychometric properties of available RCC PROMs using the methods outlined by the COSMIN group and use this as a basis for recommending PROMs for research and routine practice.^22^

## 5 DISCUSSION AND CONCLUSIONS

RCC can be life‐changing and life‐limiting, and the treatments are challenging and onerous for patients to endure and expensive for health systems and payers. Current trials are not as useful as they could be because of other reasons, such as patient selection, heterogeneity in outcome reporting, definitions and measures. A COS is needed to exploit the usefulness of future trials—to ensure the results can be compared, contrasted and synthesised where appropriate. It is important to note that there are other methodological limitations in the evidence base, such as selection and confounding biases (c.f.^7^, ^8^, ^10^, ^11^), which cannot be solved with COS development, and we acknowledge these issues will require other solutions that we do not aim to solve in this project.

Where COS have been developed in other conditions, the consistency of outcome reporting has improved. For instance, in the rheumatoid arthritis field, a COS was developed in 1994.^31^ In the intervening years, the number of trials reporting the COS has increased and is now over 80%, while selective outcome reporting has decreased.^32^

Our R‐COS will facilitate future interventional trials, help with benchmarking where appropriate and streamline the processes of systematic reviews, meta‐analyses and clinical practice guideline recommendation‐making. Ultimately, this will improve decision‐making for RCC patients, healthcare professionals, healthcare payers and policy makers.

## AUTHOR CONTRIBUTIONS

Steven MacLennan wrote the manuscript. All other co‐authors reviewed the article, provided comments and approved the article for submission.

## CONFLICT OF INTEREST STATEMENT

AB received an educational restricted grant from Pfizer for a neoadjuvant trial in kidney cancer and is a steering committee member of adjuvant kidney cancer trials at Roche/Genentech and BMS. SD is a medical advisor for Elypta. All other authors (SM, LW, KB, AL, ST, MVH, LM, RG, RW and PZ) report no conflicts of interest for this work.

## APPENDIX A COS‐STAP

### A.1

**Table:**

Title/abstract			Manuscript page
Title	1a	Identify in the title that the paper describes the protocol for the planned development of a COS.	1
Abstract	1b	Provide a structured abstract.	1
Introduction			
Background and objectives	2a	Describe the background, explain the rationale for developing the COS, and identify the reasons why a COS is needed and the potential barriers to its implementation.	3–4, for rationale; 13, for barriers to uptake and implementation strategy
	2b	Describe the specific objectives with reference to developing a COS.	5
Scope	3a	Describe the health condition(s) and population(s) that will be covered by the COS.	4
	3b	Describe the intervention(s) that will be covered by the COS.	8
	3c	Describe the context of use for which the COS is to be applied.	4
Methods			
Stakeholders	4	Describe the stakeholder groups to be involved in the COS development process, the nature of and rationale for their involvement, and also how the individuals will be identified; this should cover involvement both as members of the research team and as participants in the study.	4, stakeholders as participants 6, stakeholders as members of the research team
Information sources	5a	Describe the information sources that will be used to identify the list of outcomes. Outline the methods or reference other protocols/papers	7
	5b	Describe how outcomes may be dropped/combined, with reasons.	10
Consensus process	6	Describe the plans for how the consensus process will be undertaken.	11
Consensus definition	7a	Describe the consensus definition.	11
	7b	Describe the procedure for determining how outcomes will be added/combined/dropped from consideration during the consensus process.	11–12
Analysis			
Outcome scoring/feedback	8	Describe how outcomes will be scored and summarised, and describe how participants will receive feedback during the consensus process.	11
Missing data	9	Describe how missing data will be handled during the consensus process.	12
Ethics and dissemination			
Ethics approval/informed consent	10	Describe any plans for obtaining research ethics committee/institutional review board approval in relation to the consensus process and describe how informed consent will be obtained (if relevant)	10, ethics approvals; 11, informed consent
Dissemination	11	Describe any plans to communicate the results to study participants and COS users, inclusive of methods and timing of dissemination.	13
Administrative information			
Funders	12	Describe sources of funding and the role of funders.	14
Conflicts of interest	13	Describe any potential conflicts of interest within the study team and how they will be managed.	14
